# Temporal Trends for Patients Hospitalized With Atrial Fibrillation in the United States: An Analysis From the National Inpatient Sample (NIS) Database 2011-2018

**DOI:** 10.7759/cureus.25694

**Published:** 2022-06-06

**Authors:** Brian Foster, Adnan Liaqat, Awais Farooq, Sagar Kulkarni, Saikiran Mandyam, Pooja Patak, Mo Zaman, Paramjit Kaur, Sebastian Tosto, Arthur Kendig

**Affiliations:** 1 Internal Medicine, Southeast Health, Dothan, USA; 2 Cardiovascular Diseases, Southeast Health, Dothan, USA

**Keywords:** us health system, medical comorbidities, cost of hospitalization, length of stay, national inpatient sample database, nis, mortality rate, atrial fibrillation (af)

## Abstract

Background: Atrial fibrillation (AF) has historically been a growing burden on the global public health system. Previously, literature on the trends associated with AF-related hospitalizations has been published. However, there seems to be a gap in up-to-date information, notably within the last decade.

Purpose: This study aims to investigate the trends, outcomes, and factors associated with AF hospitalization and the continued impact of AF on the United States health system.

Methods: Patient data were collected from the years 2011 to 2018 from the National Inpatient Sample (NIS) database using the International Classification of Diseases (ICD)-9 and ICD-10 codes. We selected patients hospitalized with a diagnosis of AF. Descriptive statistics, statistical analysis, and Mann-Whitney U testing were employed to compare continuous dichotomous variables. After respective adjustments, multivariate hierarchical logistic regression was used to establish mortality rates, length of stay (LOS), and hospital charges.

Results: The study included 509,305 patients hospitalized with a primary diagnosis of unspecified AF. The mean age of patients hospitalized with AF was 71 years. AF hospitalizations were slightly higher in women as compared to men (51.7% vs. 48.2%). The predominant race involved was Caucasians at 77.9% followed by African Americans and Hispanics at 7.4% and 5.4%, respectively. The three most frequent coexisting conditions noted were hypertension (69.9%), diabetes mellitus (24.3%), and chronic obstructive pulmonary disease (16.4%). Medicare/Medicaid was the primary payer associated with the majority of AF hospitalizations at 72.6%. Overall in-hospital mortality associated with AF hospitalizations was 0.96%. Comorbid conditions conferring the highest mortality risks included coagulopathies (644%) and cerebral vascular accidents (597%). Mean LOS was found to be 3.35 days. Hospitalization charges increased year-over-year and correlated with an increase in the national burden of cost for these patients of $3.6 billion.

Conclusions: Our study investigates the national trends surrounding AF hospitalizations. Overall in-hospital mortality rates appear to be stable as compared to prior years and past literature. Comorbid conditions conferring significantly higher mortality rates included coagulopathies, cerebral vascular accidents, acute kidney injury, and end-stage renal disease. Additionally, suboptimal insurance status was also associated with increased mortality risk. The cost of hospitalization in AF patients has increased steadily, conferring a $3.6 billion burden on the US healthcare system.

## Introduction

Atrial fibrillation (AF) has historically been a growing burden on the global public health system [1]. The rising incidence and prevalence of AF in the United States continue to stress the public from both a medical and economic standpoint [1,2]. In developed nations, the number of men and women affected by AF is projected to double over the next two decades [3]. The prevalence of AF is projected to increase to 15.9 million by the year 2050 [4]. AF is notably associated with stroke, structural heart disease, congestive heart failure, and impaired quality of life [5-7]. This results in significant demand on the healthcare system, along with a substantial economic burden. Previously, literature on the trends associated with AF-related hospitalizations has been published [4,8-10]. However, there seems to be a gap in up-to-date information, most notably within the last decade. To continue to support the national healthcare system, it is imperative that researchers and clinicians stay diligent in their understanding of the current trends associated with AF-related hospitalizations. This study aims to identify particular patterns surrounding AF-related hospitalizations across its various subpopulations, association with various comorbid diagnoses, in-hospital mortality rates, impact on the length of stay (LOS), and updated understanding surrounding current trending AF-related billing and coding.

## Materials and methods

The National Inpatient Sample (NIS) of the Healthcare Cost and Utilization Project (HCUP), sponsored by the Agency for Healthcare Research and Quality (AHRQ), was used for this study [11]. It is the largest inpatient hospitalization-related healthcare dataset in the United States. NIS includes data from all states taking part in HCUP, which approximates 97% of the US population (NIS Database Documentation) [11]. It utilized billing International Classification of Diseases (ICD) codes to confer patient diagnoses while supplying socioeconomic data. It represents a 20% sample from inpatient hospitalizations every year. Patient data were collected for the years 2011-2018 from this database and we identified hospitalized patients with a documented diagnosis of unspecified AF in US hospitals using ICD-9 and ICD-10 codes in congruence with previously widely accepted literature [4]. Descriptive statistics were used to summarize the baseline characteristics of our patient population. We compared baseline demographics including age, sex, race, geographic location, and primary expected payer in AF-related hospitalizations. Patients younger than 18 years were excluded. The primary outcome was AF-related in-hospital mortality. Secondary outcomes were comorbid conditions, socioeconomic factors associated with AF, mean LOS, and total hospitalization charges ($). Statistical analysis was performed with Mann-Whitney U testing to compare continuous dichotomous variables. After adjusting for hospital size, type, and transfer status, a hierarchical multivariate regression model was used to calculate adjusted odds ratios (AOR) with 95% confidence intervals (CIs). P-value < 0.05 was considered significant. We used IBM SPSS Statistics 28.0 software (IBM Corp., Armonk, NY) for all statistical analysis.

## Results

AF hospitalizations, demographics, and comorbidities

Our study included a total of 509,305 patients hospitalized with a primary diagnosis of unspecified AF in the United States from 2011 to 2018. Patient characteristics are summarized in Table 1. The mean age of patients hospitalized with AF was 71 years, with the largest age group of 18-65 years. AF hospitalizations were slightly higher in women (51.7%) versus men (48.2%). This trend was seen throughout each year; however, the difference between sexes decreased notably to less than 1000 patients through the years 2016, 2017, and 2018. The predominant race seen in AF patients was Caucasian (77.9%), followed by African Americans (7.4%) and Hispanics (5.4%). African Americans and Hispanics saw an increase in AF hospitalizations by over 1.5% throughout 2016, 2017, and 2018, while Caucasians saw a decrease in AF hospitalizations of 2-3% throughout the same period. The three most frequent coexisting conditions in patients hospitalized with AF were noted to be hypertension (69.9%), diabetes mellitus (24.3%), and chronic obstructive pulmonary disease (COPD) (16.4%). The prevalence of multiple comorbidities in AF hospitalizations significantly changed throughout the years from 2011 to 2018. Acute kidney injury (AKI) had the largest increase in prevalence in AF hospitalized patients in 2018 versus 2011 (12.64% vs. 6.37%, respectively). Diabetes mellitus had the largest decrease in prevalence in AF hospitalized patients (23.95% vs. 19.63%) in 2018 versus 2011. Medicare/Medicaid was the primary payer associated with the majority (72.6%) of AF hospitalizations.

**Table 1:** Baseline characteristics of patients hospitalized for a primary diagnosis of atrial fibrillation subdivided by mortality

		No mortality		Mortality		Total	
		Mean	Count	Mean	Count	Mean	Count
Age in years at admission		70.45 years		78.55 years		70.53 years	
Age groups	18-34 years		7,165		13		7,178
	35-49 years		29,691		66		29,757
	50-64 years		119,736		548		120,284
	65-79 years		198,867		1,570		200,437
	80 years and older		148,633		2,732		151,365
Sex	Male		243,362		2,106		245,468
	Female		260,671		2,823		263,494
Race	Caucasian		393,066		3,817		396,883
	African American		37,582		401		37,983
	Hispanic		27,402		254		27,656
	Asian or Pacific Islander		6,833		83		6,916
	Native American		1,786		19		1,805
	Other		9,970		85		10,055
Hypertension	No		151,365		1,850		153,215
	Yes		352,727		3,079		355,806
Chronic kidney disease	No		436,779		3,722		44,0501
	Yes		67,313		1,207		68,520
End-stage renal disease	No		492,889		4,581		497,470
	Yes		11,203		348		11,551
Diabetes	No		381,031		3,828		384,859
	Yes		123,061		1,101		124,162
Dyslipidemia	No		266,708		3,181		269,889
	Yes		23,738		1,748		239,132
Tobacco use	No		450,286		4,538		454,824
	Yes		53,806		391		54,197
Chronic obstructive pulmonary disease	No		421,977		3,522		425,499
	Yes		82,115		1,407		83,522
Obstructive sleep apnea	No		453,538		4,679		458,217
	Yes		50,554		250		50,804
Peripheral vascular disease	No		483,441		4,575		488,016
	Yes		20,651		354		21,005
Obesity	No		454,633		4,718		459,351
	Yes		49,459		211		49,670
Aortic valve disorder	No		484,528		4,655		489,183
	Yes		19,564		274		19,838
Mitral valve disorder	No		460,620		4,541		465,161
	Yes		43,472		388		43,860
Tricuspid valve disorder	No		500,942		4,902		505,844
	Yes		3,150		27		3,177
Pulmonic valve disorder	No		502,955		4,918		507,873
	Yes		1,137		11		1,148
Transient ischemic attack	No		501,485		4,904		506,389
	Yes		2,607		25		2,632
Cerebrovascular disease	No		501,509		4,749		506,258
	Yes		2,583		180		2,763
Coagulopathy	No		502,841		4,822		507,673
	Yes		1,241		107		1,348
Acute kidney injury	No		460,487		3,301		463,788
	Yes		43,605		1,628		45,233

In-hospital mortality

Overall in-hospital mortality associated with AF hospitalizations was less than 1% (0.96%). Initially, a rate of less than 1% was seen from the years 2011 to 2015. This rate increased to above 1% in years 2016, 2017, and 2018 (1.18%, 1.17%, and 1.10%, respectively). The mortality rate was highest in patients aged 85 years and older (2.19%). Comorbid conditions that conferred the highest mortality risk included coagulopathies (644%, OR: 6.442, CI: 5.179-8.013, P < 0.05) and cerebral vascular accidents (CVA) (597%, OR: 5.975, CI: 5.063-7.052, P < 0.05). Medicare/Medicaid was again the primary payer associated with the highest in-hospital mortality rates (1.92%). The mean LOS in patients who died was 6.57 days. The mean hospitalization cost for patients who died was $70,033.64.

Length of stay, cost of hospitalization, and disposition

LOS, cost of hospitalization, and primary expected payer findings subdivided by mortality are summarized in Table 2. The mean LOS was found to be 3.35 days in patients hospitalized with AF. The mean hospitalization cost for the years 2011 to 2018 was $33,402.77. This number steadily increased year by year, from $30,066.83 in 2011 to $37,145.75 in 2018. This correlates to an increase in the national burden of cost in AF hospitalizations of $3.6 billion from 2011 to 2018 ($15.3 billion in 2011 vs. $18.9 billion in 2018). Disparities among dispositions were noted, as seen in Table 3. African Americans had a 27.6% higher chance of non-routine discharge (against medical advice, nursing home, skilled nursing facility, long-term acute care hospital, etc.). The female sex was noted to have a 28.9% higher chance of non-routine discharge. Among comorbidities, CVA conferred the highest risk of non-routine discharge at 528%, followed by coagulopathy at 207.3% and AKI at 199.8%. Finally, larger bed-size hospitals were known to confer a smaller risk of non-routine discharge (Table 4).

**Table 2:** Multivariate hierarchical logistic regression for associated risk factors in patients hospitalized with atrial fibrillation and their mortality risk Coagulopathies, cerebral vascular accidents, and end-stage renal disease conferred the highest mortality risk amongst atrial fibrillation patients.

	Significance	Odds ratio	95% confidence interval for odds ratio	
Hypertension	<0.001	0.653	0.613	0.615
Chronic kidney disease	0.170	1.056	0.977	1.141
End-stage renal disease	<0.001	4.504	3.978	5.101
Diabetes	0.003	0.894	0.830	0.963
Dyslipidemia	<0.001	0.645	0.605	0.688
Tobacco use	0.416	0.953	0.847	1.071
Chronic obstructive pulmonary disease	<0.001	1.786	1.668	1.913
Obstructive sleep apnea	<0.001	0.749	0.652	0.860
Peripheral vascular disease	<0.001	1.318	1.172	1.483
Obesity	<0.001	0.672	0.580	0.779
Aortic valve disorder	0.649	1.031	0.905	1.173
Mitral valve disorder	0.002	0.842	0.754	0.941
Tricuspid valve disorder	0.042	0.654	0.434	0.985
Pulmonic valve disorder	0.989	0.996	0.545	1.818
Transient ischemic attack	0.322	0.807	0.527	1.235
Cerebrovascular accident	<0.001	5.975	5.063	7.052
Anemia	<0.001	1.313	1.204	1.433
Coagulopathy	<0.001	6.442	5.179	8.013
Acute kidney injury	0.000	4.179	3.895	4.482

**Table 3:** Length of stay, cost of hospitalization, and primary expected payer among patients hospitalized with a primary diagnosis of atrial fibrillation subdivided by mortality Note significantly increased mean length of stay and mean cost of hospitalization amongst atrial fibrillation patients who suffered mortality.

		No mortality	Mortality	Total
Mean length of stay		3.35 days	6.57 days	3.38 days
Mean cost of hospitalization		$32,370.12	$70,033.64	$32,734.08
Primary expected payer	Medicare	339,386	4,047	343,433
	Medicaid	26,040	193	26,233
	Private insurance	111,681	474	112,155
	Self-pay	14,838	77	14,915
	No charge	1,542	6	1,548
	Other	9,838	124	9,962

**Table 4:** Multivariate hierarchical logistic regression exploring associated risk factors for non-routine discharge

	Significance	Odds ratio	95% confidence interval for odds ratio	
Hypertension	2.147E-32	0.0905	0.890	0.920
Chronic kidney disease	2.356E-175	1.343	1.315	1.370
End-stage renal disease	1.623E-192	1.951	1.867	2.040
Diabetes	1.406E-197	1.296	1.274	1.318
Dyslipidemia	7.989E-262	0.768	0.756	0.779
Tobacco use	0.000	1.070	1.042	1.099
Chronic obstructive pulmonary disease	0.000	1.653	1.622	1.684
Obstructive sleep apnea	0.281	0.985	0.959	1.012
Peripheral vascular disease	7.343E-38	1.242	1.201	1.283
Obesity	4.250E-35	0.841	0.818	0.865
Aortic valve disorder	3.931E-25	1.194	1.155	1.235
Mitral valve disorder	0.575	0.993	0.968	1.018
Tricuspid valve disorder	0.000	1.185	1.088	1.289
Pulmonic valve disorder	0.248	0.917	0.792	1.062
Transient ischemic attack	0.000	1.266	1.156	1.387
Cerebrovascular accident	3.992E-273	5.286	4.819	5.798
Anemia	0.00	1.135	1.068	1.206
Coagulopathy	2.836E-28	2.073	1.821	2.359
Acute kidney injury	0.000	1.998	1.950	2.046
Indicator of sex	3.850E-233	1.289	1.270	1.309
African American race	5.847E-65	1.276	1.241	1.313
Hispanic race	0.000	0.940	0.909	0.971
Asian or Pacific Islander race	0.000	0.849	0.796	0.904
Native American race	0.002	0.817	0.719	0.928
Other race	0.000	1.099	1.043	1.157

## Discussion

Our study highlights up-to-date data surrounding the current state of AF-related hospitalizations in the United States. Overall in-hospital mortality of AF hospitalizations appears unchanged [4,8]. We did observe in-hospital mortality rates to substantially increase over the more recent years (2016-2018). However, during the same time frame, a significant drop in patients with a primary diagnosis of AF was also noted. This decrease in the patient population may have falsely increased the mortality rate. Women continue to suffer from AF-related hospitalizations at a higher rate than men [8-10]. However, there was no statistically significant difference in mortality outcomes. In regards to age, previous findings seem to be replicated, as increased in-hospital mortality rates were seen with increased age [12].

The three most common comorbidities seen with AF hospitalizations continue to be hypertension, diabetes, and COPD [4,6,13]. Obstructive sleep apnea was additionally prevalent among our patient population, signifying the risk conferred from pulmonary pathology contributing to AF hospitalization. The highest mortality rates in AF hospitalizations were seen in patients also suffering from coagulopathies and CVA. End-stage renal disease (ESRD) and AKI additionally conferred significantly higher mortality risk. The latter may be explained by patients who present with AF with a rapid ventricular response and concurrent hemodynamic compromise, worsening their overall prognosis. Coagulopathies and CVA may have increased mortality risk among our patients due to possible supratherapeutic anticoagulation or failed anticoagulation therapy leading to eventual development or worsening of CVA. However, NIS does not afford enough granular detail to determine various pharmacotherapies associated with each patient's hospitalization.

Current projections expect the national health expenditures to grow by an overall rate of 5.4% for the years 2019-2028 [14]. Previous literature cites mean hospitalization costs in the $6000-$8000 range [4,15]. We found the mean hospitalization cost to be substantially higher than previously described (upwards of $30,000). Furthermore, mean hospitalization cost was found to increase roughly $7,000 over the same timeframe. Part of this increase may be explained by increased LOS, a mean of 3.35 days in our study versus less than three days in prior studies [4]. However, although we did observe an increase in LOS, due to differences in our underlying patient population, we are unable to draw specific conclusions due to hospital differences in patient complexity.

We observed socioeconomic factors modulating mortality risk as well. Patients with Medicaid as a primary payer had a 63% increased risk of mortality compared to Medicare. Self-pay, no charge, and other payer statuses also conferred a higher risk of mortality. This illustrates that suboptimal insurance status is associated with increased mortality risk. We believe these differences may also play a role in explaining increased risk in mortality with coagulopathies, as patients with suboptimal insurance status are more prone to be on warfarin therapy, thus increasing the risk for bleeding complications. Finally, private pay patients were noted to have a 49% lower risk of non-routine discharge when compared to Medicaid and Medicare patients, possibly indicating an initially healthier patient population.

Limitations

Although our study utilizes a large, well-known administrative database, it is not without its limitations. The primary diagnosis code ran was unspecified AF. It is entirely possible that patients presenting with and admitted for other reasons could qualify for our study, however, they had a different diagnosis coded in the primary position, thus excluding them from our study population. Furthermore, although we investigated the use of other more specified AF codes (notably paroxysmal AF, other persistent AF, and long-term persistent AF), we did not factor these patients into our original/total patient population. Essentially, we only included patients hospitalized with a primary diagnosis of unspecified AF, keeping in congruence with prior published studies [4]. Moreover, we sought to specifically focus on patients without a previously established diagnosis of AF. We believe utilizing a primary diagnosis of unspecified AF was the best way to accomplish this. Thus, when comparing our study to existing literature, the study’s sample size and subsequent power may be less than previous studies [3]. Additionally, the NIS is known for logging each hospitalization as its own entry. Thus, it is impossible to separate index cases from readmissions. Even more so, many patients admitted with AF originally present with AF with a rapid ventricular response; a diagnosis that does not have a specific corresponding ICD-9 or ICD-10 code. Lastly, because our study investigated AF hospitalizations and does not include outpatient data or long-term follow-up outcomes, overall incidence, prevalence, and mortality rates may be underestimated. However, despite the aforementioned limitations, our study offers some strengths when compared to previous literature on the same topic: updated data for years not included in prior studies, a more specific study population with a primary diagnosis of AF, and improved analytics surrounding causal associations given logistical regression analysis.

It must be noted that the patient population experienced a significant drop-off from 2016 to 2018. This was originally felt to be due to more precise diagnosis coding; however, after investigating more specific ICD-10 codes surrounding AF (paroxysmal, persistent, long-standing persistent, and permanent), an additional 97 patients were found. Thus, the initial drop in patients hospitalized for unspecified AF cannot be explained by the above. One explanation may be the utilization of comorbid diagnosis in the primary position to increase hospitalization reimbursement rates with the coding of unspecified AF in subsequent positions.

## Conclusions

Here, we report updated data surrounding trends of patients suffering AF hospitalization. Overall in-hospital mortality rates appear stable when compared to prior studies. Comorbid conditions conferring significantly higher mortality rates included coagulopathies, CVA, AKI, and ESRD. Suboptimal insurance status was also associated with increased mortality risk. The cost of hospitalization has steadily increased year over year. It is imperative that continued efforts to both prevent and manage AF hospitalizations be developed, studied, and refined to reduce the burden of AF on the US healthcare system.
